# DisGeNet: a disease-centric interaction database among diseases and various associated genes

**DOI:** 10.1093/database/baae122

**Published:** 2025-01-11

**Authors:** Yaxuan Hu, Xingli Guo, Yao Yun, Liang Lu, Xiaotai Huang, Songwei Jia

**Affiliations:** School of Computer Science and Technology, Xidian University, 266 Xinglong Section of Xifeng Road, Xi’an, Shaanxi 710126, China; School of Computer Science and Technology, Xidian University, 266 Xinglong Section of Xifeng Road, Xi’an, Shaanxi 710126, China; School of Computer Science and Technology, Xidian University, 266 Xinglong Section of Xifeng Road, Xi’an, Shaanxi 710126, China; School of Computer Science and Technology, Xidian University, 266 Xinglong Section of Xifeng Road, Xi’an, Shaanxi 710126, China; School of Computer Science and Technology, Xidian University, 266 Xinglong Section of Xifeng Road, Xi’an, Shaanxi 710126, China; School of Computer Science and Technology, Xidian University, 266 Xinglong Section of Xifeng Road, Xi’an, Shaanxi 710126, China

## Abstract

The pathogenesis of complex diseases is intricately linked to various genes and network medicine has enhanced understanding of diseases. However, most network-based approaches ignore interactions mediated by noncoding RNAs (ncRNAs) and most databases only focus on the association between genes and diseases. Based on the mentioned questions, we have developed DisGeNet, a database focuses not only on the disease-associated genes but also on the interactions among genes. Here, the associations between diseases and various genes, as well as the interactions among these genes are integrated into a disease-centric network. As a result, there are a total of 502 688 interactions/associations involving 6697 diseases, 5780 lncRNAs (long noncoding RNAs), 16 135 protein-coding genes, and 2610 microRNAs stored in DisGeNet. These interactions/associations can be categorized as protein–protein, lncRNA–disease, microRNA–gene, microRNA–disease, gene–disease, and microRNA–lncRNA. Furthermore, as users input name/ID of diseases/genes for search, the interactions/associations about the search content can be browsed as a list or viewed in a local network-view.

**Database URL**: https://disgenet.cn/

## Introduction

Complex diseases typically result from the combined effects of multiple genes and various environmental factors [1]. The onset and progression of these diseases can vary significantly between individuals. For example, the rate at which a patient’s condition progresses can differ greatly. In addition, the prevalence of certain complex diseases is influenced by factors such as gender, with autoimmune diseases showing a much greater prevalence on women [2]. Therefore, studying complex diseases solely from a single-factor perspective may not provide a comprehensive understanding of their complexity. As regards to the disease-associated genes, not only protein-coding genes but also noncoding genes along with the interactions among them can give a more thorough perspective on complex diseases. In recent years, with the development of network medicine, it has offered quantitative insights into disease mechanisms, comorbidities, as well as novel diagnostic tools and treatment methods [3].

From the perspective of network medicine, most studies of diseases rely on a comprehensive map of protein–protein interactions (PPIs). For example, IQSEC2 is a protein associated with neurons, and its interaction with the guanine nucleotide-binding (GTP-binding) protein ARF6 may play a crucial role in conditions such as autism and intellectual disabilities [4]. However, the onset and development of diseases are influenced by interactions among various genes. There is overwhelming evidence that ncRNAs regulate multiple biological processes, playing important roles in multiple diseases. For example, lncRNA–microRNA regulates cell death mechanisms such as apoptosis and autophagy in cancers [5]. Besides, the study in [6] suggests that *miR-122a* and *miR-422a* may destabilize *CYP7A1* mRNA to inhibit *CYP7A1* expression which plays a critical role in regulation of bile acid synthesis in the liver. Therefore, to enhance the understanding of disease mechanisms in network medicine, interactions mediated by ncRNAs must be incorporated into the framework of network medicine.

As a disease-centric view, the various genes (such as protein-coding genes, lncRNAs, and microRNAs) play a crucial regulatory role in the occurrence and development of complex diseases. Several databases are devoted to categorize these associations, such as the associations between diseases and protein-coding genes, the associations between diseases and microRNAs, also the associations between the diseases and lncRNAs. The current version of Online Mendelian Inheritance in Man (OMIM) [7] contains 27 140 entries and the OMIM Morbid Map Scorecard contains 7439 molecularized phenotypes connected to 4852 genes. Additionally, LncRNADisease [8] comprises 25 440 ncRNA–disease associations across 6066 lncRNAs, 10 732 circRNAs, and 566 different diseases. Among these, there are 13 191 experimentally supported lncRNA–disease associations. Moreover, Human MicroRNA Disease Database (HMDD) [9] curates 53 530 microRNA–disease association entries, which include 1817 human microRNA genes, 79 virus-derived microRNAs, and 2360 diseases from 37 090 papers. However, disease is associated with various genes, yet these databases only focus on the associations between specific genes and genes, without integrating connections between multiple genes and diseases.

**Figure 1.**
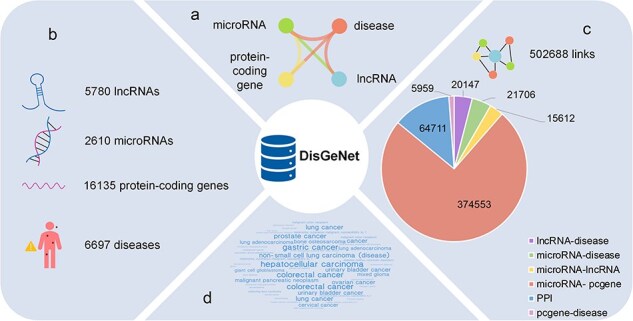
The statistics of DisGeNet: (a) six types of links network, (b) four types of nodes, (c) statistics of links, and (d) the top 50 diseases associated with genes.

Taken together, in order to achieve a more comprehensive understanding of diseases, we have developed a comprehensive database called DisGeNet, which incorporates interactions mediated by ncRNAs and captures a diverse array of links among the diseases and their associated genes, visually depicting the intricate associations between diseases and genes, as well as the interactions among distinct genes in a network perspective. As shown in Fig. 1A, the basic information in the network, including four types of nodes and six types of links, is displayed.

## Materials and methods

### Data resources

DisGeNet curates widely recognized data sources that align with its disease-centric theme, focusing on association of genes with diseases, as well as the interactions among these related genes.

When it comes to disease data, MONDO [10] provides a comprehensive disease ontology that integrates multiple disease-data sources, offering detailed disease classification suitable for cross-database comparison and analysis. Disease Ontology (DO) [11] delivers standardized disease classification and disease–gene association data. Medical Subject Headings (MeSH) [12], maintained by the US National Library of Medicine, supports biomedical literature indexing with its controlled vocabulary. While OMIM [7] focuses on human genes and genetic disorders, providing extensive information on genes and their associated diseases.

For protein-coding gene and lncRNA data, HUGO Gene Nomenclature Committee (HGNC) [13] supplies standardized nomenclature for human genes, ensuring consistency and traceability. The reference gene annotation for human and mouse (GENCODE) [14] provides accurate gene feature identification and classification in human and mouse genomes, and NCBI Gene [15] includes data across species, covering nomenclature, reference sequences, and phenotypes. Additionally, comprehensive data on both protein-coding gene and lncRNA are provided by these databases.

With regard to microRNA data, miRBase [16] encompasses extensive coverage, including microRNA sequences, structural predictions, genomic locations, and expression data across various species.

Furthermore, concerning lncRNA–disease associations, Lnc2Cancer [17] offers information on lncRNA–circRNA associations with human cancers, supported by experimental evidence. RNADisease [18] covers both literature-validated and predicted RNA–disease interactions. LncRNADisease [8] integrates comprehensive data from manual literature curation and other sources, providing a solid basis for research into lncRNA–disease associations. For microRNA–disease associations, HMDD [9] provides its collection of experimentally supported microRNA–disease associations, making it a valuable reference for researchers in the field. Regarding protein-coding gene–disease associations, OMIM [7] provides its expert-reviewed content, offering extensive information on gene–disease associations with detailed descriptions of monogenic genetic disorders, including pathological mechanisms, inheritance patterns, and clinical features.

As for microRNA–protein-coding gene interactions, miRTarBase [19] encompasses over 360 000 miRNA–protein-coding gene interactions, curated through a combination of manual review and automated processes to ensure the inclusion of the latest research. Besides, STRING [20] offers rigorously validated and scored data on PPIs, derived from multiple high-quality studies, encompassing both known and predicted interactions, including direct (physical) and indirect (functional) interactions. Lastly, ENCORI [21] is an advanced platform for microRNA–lncRNA interactions, providing an integrative display of RNA–RNA and protein–RNA interactions. It utilizes sophisticated software and pipelines to analyze high-throughput sequencing data, presenting interaction data and detailed functional annotations. A comprehensive review of the databases providing the aforementioned data types is detailed in the Supplementary Table S1.

### Nodes in DisGeNet

As for diseases, 23 234 entries from MONDO (as of 4 July 2024) [10], 14 082 entries from DO (as of 4 July 2024) [11], 30 724 Main Heading Terms and 323 744 Supplementary Concept Record Terms from MeSH (as of 4 July 2024) [12], and 26 820 entries from OMIM (as of 30 July 2020) [7] are extracted with key information such as IDs, names, and synonyms. Because MeSH is primarily focused on medical subject terms found in MEDLINE/PubMed, NLM catalogs and other NLM databases, it is suitable for medical subject retrieval [12]. OMIM specializes in the molecular relationships between genetic variations and phenotypic expressions, providing detailed information on genetic diseases and associated genes [7]. There is some overlap between DO and MeSH or OMIM, and conflicts may arise in mapping. However, MONDO provides a logic-based framework for integrating multiple disease resources, allowing it to retain a broader range of disease entries [10]. Therefore, MONDO is selected as the primary reference data source, and information from other sources is used to supplement it, ultimately resulting in 23 234 disease entries with detailed information.

Concerning protein-coding genes and lncRNAs, 16 260 entries for protein-coding genes and 5765 entries for lncRNAs in HGNC (as of 4 July 2024) [13] are downloaded. Besides, annotation files are first downloaded from GENCODE46 [14], from which key information such as various IDs names, gene types, and transcript IDs are extracted. Subsequently, genes with the same gene ID are merged and duplicates are removed, resulting in 20 065 protein-coding gene entries and 19 258 lncRNA entries. After filtering human data, 20 688 protein-coding gene entries and 20 188 lncRNA entries are extracted from NCBI Gene (as of 4 July 2024) [15]. Finally, the data from the three sources are merged using outer joins, missing values are filled with nulls, and gene type and synonyms information are deleted, resulting in a final dataset of 21 291 protein-coding gene entries and 32 839 lncRNA entries.

**Figure 2.**
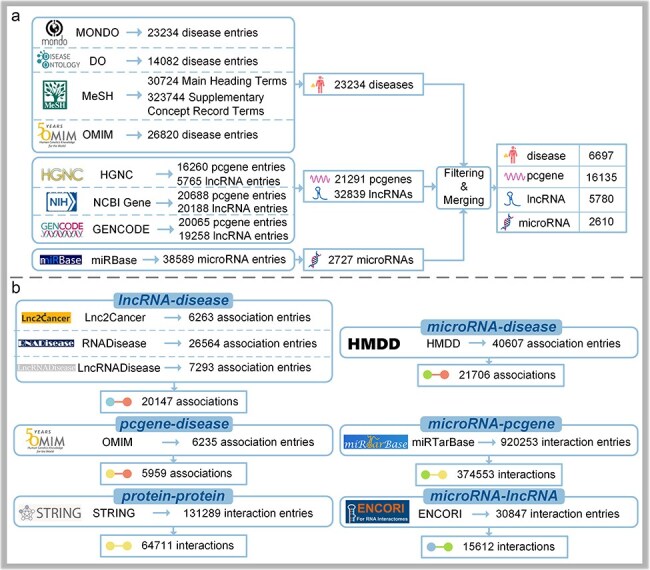
The data collection process of DisGeNet: (a) the collection of node information and (b) the collection of link information.

With regard to microRNAs, there is a category of 38 589 microRNA entries in miRBase22.1 [16]. We focus on human microRNAs; meticulous curation and filling in missing IDs with aliases are explored, leading to a final set of 2727 mature microRNA entries. Details are illustrated in Fig. 2A.

### Links in DisGeNet

As for lncRNA–disease associations, 6263 entries from Lnc2Cancer3.0 [17], 26 564 entries from RNADisease4.0 [18], and 7293 entries from LncRNADisease3.0 [8] are extracted by filtering for human data and mapping against the previously obtained lncRNA and disease datasets. Subsequently, merging using outer join, filling missing values with nulls and removing duplicate rows are performed on these associations, resulting in a standardized dataset comprising 20 147 lncRNA–disease associations.

Concerning microRNA–disease associations, there is a category of 40 607 entries in HMDD4.0 [9]. We focus on the obtained datasets of diseases and microRNAs, and the mapping results are retained with relevant information filled in. Finally, a set of 21 706 microRNA–disease associations in human are curated in DisGeNet.

With regard to protein-coding gene–disease associations, 6235 entries from OMIM [7] are downloaded as of 30 July 2020. These entries are then matched based on the previously obtained protein-coding gene and disease datasets. After data organization and removal of duplicate entries are performed, a total of 5959 protein-coding gene–disease associations are provided in DisGeNet.

As for PPI data, 131 289 human-related PPIs are downloaded from STRING12.0 [20] and mapped against the obtained protein-coding gene file, with the mapping results retained and relevant information filled in, resulting in 64 711 PPIs.

**Figure 3.**
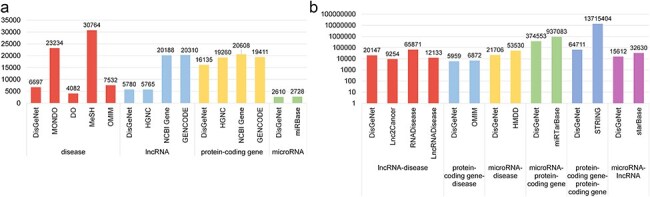
Statistical comparison of data in DisGeNet: (a) node data and (b) link data.

Similarly, a total of 374 553 microRNA–protein-coding gene interactions and 15 612 microRNA–lncRNA interactions are obtained by separately downloading 920 253 interactions and 30 847 interactions from miRTarBase9.0 [19] and ENCORI [21], and mapping them against the previously obtained microRNA and protein-coding gene datasets, as well as microRNA and lncRNA datasets. Details are illustrated in Fig. 3B.

### Data integration

Based on the processed link data, filtering of node information with associated links and merging of duplicates with matching primary identifiers are performed (Fig. 2A), resulting in 5780 lncRNA entries, 16 135 protein-coding gene entries, 2610 microRNA entries, and 6697 disease entries.

After all these preprocessing, as shown in Fig. 1B and C, 6697 disease nodes, 5780 lncRNA nodes, 16 135 gene nodes, and 2610 microRNA nodes are provided in DisGeNet, and the detailed information for these nodes are curated carefully in our database, which is shown in Supplementary Table S2. Besides, the 502 688 links encompass 20 147 lncRNA–disease associations, 21 706 microRNA–disease associations, 5959 protein-coding gene–disease associations, 374 553 microRNA–protein-coding gene targeting interactions, 15 612 microRNA–lncRNA targeting interactions, and 64 711 PPIs. Among them, there are 379 189 overlapping data between gene–gene interactions and Barabási Laboratory’s research on network medicine [3]. The information of the links in our database is described in Supplementary Table S3.

## Results

### Technical implementation of DisGeNet

DisGeNet is developed with HTML and Python language using MySQL (http://www.mysql.com/) as the database management system. The web interface is developed based on the Bootstrap (http://www.getbootstrap.com/3.3.4/) framework and JavaScript. To support the growing user base, we optimize the database querying algorithms and implement partitioning and indexing techniques to enhance query efficiency and response times. Additionally, stress testing is conducted to ensure the DisGeNet’s stability and performance under high concurrency conditions.

### Comparative analysis and predictions of data

As illustrated in Fig. 3, we have conducted a comparative analysis of node and edge data statistics from DisGeNet and other databases. While the amount of data in DisGeNet may be relatively smaller compared to other databases, this is due to DisGeNet’s rigorous selection of data on disease-associated genes and interactions among these genes, ensuring the reliability and usability of the information. This comprehensive coverage highlights DisGeNet’s advantage in integrating data across multiple biological categories.

Additionally, data from DisGeNet is utilized to predict potential genes associated with diseases, particularly ncRNAs, allowing for a deeper understanding and analysis of disease mechanisms from a disease-centric perspective. First, the disease node is focused on, and subgraph data related to its associated genes are downloaded. Subsequently, multiple subgraph datasets for each related gene are downloaded, representing their links with other diseases/genes. By merging these subgraph datasets and performing link prediction, potential associations between the disease and genes not yet included in the DisGeNet database are validated through literatures. Using non-small cell lung cancer (NSCLC) as an example of a high-degree disease node, potential links between NSCLC and genes such as *KRAS*, *LINC00963*, and *MIR17HG* are discovered. Among them, *LINC00963* and *MIR17HG* are lncRNAs, and the significant roles of these genes in the onset and progression of NSCLC have been substantiated by previous studies, including those by Principe et al. [22], Xie et al. [23], and Zheng et al. [24]. Similarly, for a disease node with a lower degree, such as kidney cancer, potential association with the lncRNA *NEAT1* is identified. Research conducted by Liu [25] has demonstrated that *NEAT1* promotes the proliferation and migration of kidney cancer cells. These findings further confirm the reliability and usability of DisGeNet. The detailed information of these predicted links is presented in Supplementary Table S4.

### Web interface

**Figure 4.**
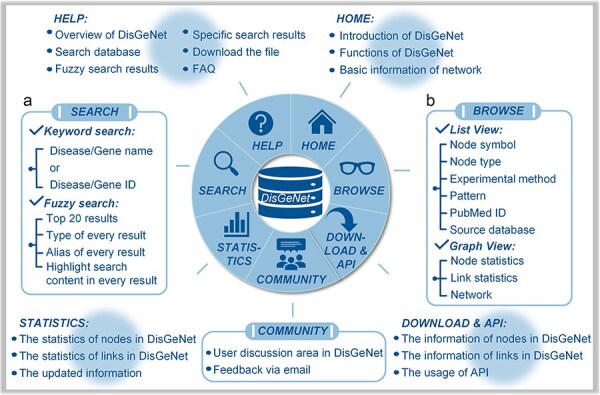
Interface of DisGeNet: (a) search for all associations and interactions, and (b) browse all associations and interactions.

**Figure 5.**
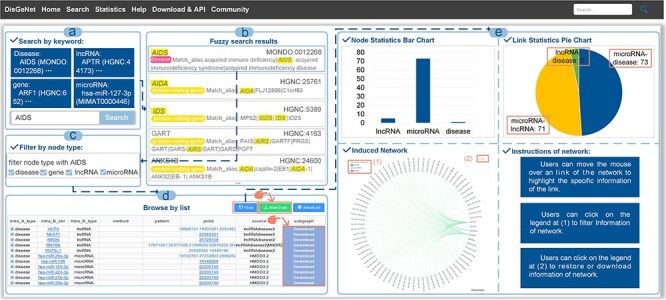
A general use process of DisGeNet: (a) the interface of the search module with “AIDS” input as the retrieved disease in the “Search” page (disease name used as an example), (b) fuzzy search result page of “AIDS”, (c) filtering links by node types, (d) the search results shown in a list manner, and (e) the search results shown in a graph manner.

The “Search” page provides “keyword search” and “fuzzy search” (Fig. 4A). In keyword search page, users can search by name/ID of gene/disease such as MONDO_ID of disease, miRBase_ID of microRNA and HGNC_ID, Ensembl_ID or NCBI GeneID of lncRNA, and protein-coding gene (Fig. 5A). In fuzzy search, the search results will be limited to displaying only the top 20 results that are most relevant or similar. Additionally, in each search result, the parts that are similar to the search query will be highlighted, facilitating users to locate the target search content (Fig. 5B).

With regard to the search results, the links among the genes, input disease or gene and the associated other diseases can be browsed in a manner of “List” or “Graph,” as shown in Fig. 4B. In the list manner, users can filter the given links by node type to obtain the specified retrieval (Fig. 5C). Furthermore, detailed information about the links, such as node symbols, node types, experimental methods used, interaction patterns, the PubMed ID (PMID) of reference articles, and the source database is provided in the list. Users also can download the association subgraph centered on a specific disease or gene by clicking the “Download” button (Fig. 5D). In the graph manner, statistics for nodes and links, as well as detailed information about the induced subnetwork, are visualized in a graph view. In addition, users can click on node types to hide the corresponding type of links in “Subnetwork Graph.” Finally, when users move the mouse cursor in “Node Statistics Bar Chart,” “Link Statistics Pie Chart,” and “Subnetwork Graph,” they can highlight specific data and access its detailed information (Fig. 5E). The detailed search example of DisGeNet is illustrated in the Supplementary Data.

**Figure 6.**
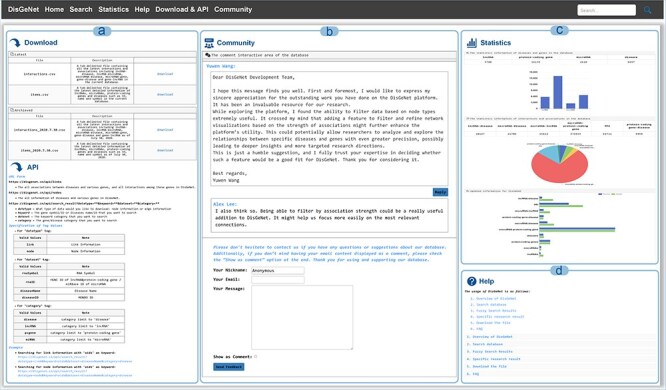
Other functions in DisGeNet: (a) get data via “Download” or “API,” (b) discussion and feedback in DisGeNet, (c) data statistics in DisGeNet, and (d) learn more about DisGeNet through the “Help” interface.

On the “Download & API” page, users can access both current and historical database data, as well as efficiently retrieve and query biological data via the API interface. The API allows users not only to access all node and edge information but also to perform parameterized queries to precisely download node and edge data related to specific diseases or genes (Fig. 6A).

From the “Community” page, users are able to submit feedback via email, submit comments or suggestions, and participate in discussions (Fig. 6B). The “Statistics” page, the data statistics for each type and a comparison of the data before and after updates of DisGeNet are presented in the form of charts (Fig. 6C). For additional information and further instructions, users can refer to the “Help” page (Fig. 6D).

## Discussion and future extensions

An increasing body of evidence indicates that the association between diseases and genes, as well as the interactions among these associated genes, plays a crucial role in understanding diseases. Moreover, by including ncRNAs into the PPIs, it is possible to retrieve more complete and biologically more meaningful list of disease–disease interactions, offering a better quantitative understanding of disease similarity and comorbidity, ultimately helping us understand disease progression in patients. Therefore, DisGeNet, a disease-centric database, integrates ncRNAs into network medicine and encompasses associations between diseases and various types of genes, as well as interactions among different genes.

To ensure the relevance and accuracy of the information, DisGeNet is updated on an annual basis. This regular update schedule allows us to promptly incorporate changes from source databases and continuously enhance the database’s gene prediction capabilities. As research continues to advance, an increasing number of gene types associated with diseases, such as circRNA, piRNA, and eRNA, are being discovered. As more comprehensive data becomes available, we plan to integrate these gene types into our database. Therefore, DisGeNet provides a global network view to understand the mechanisms of complex diseases based on all these links among the associated genes. With accumulating disease–gene associations and interactions among various genes, we will have a deeper and more comprehensive understanding of the disease based on all these links. To sum up, DisGeNet is just a pioneering platform in network medicine that will serve as a valuable resource for researchers studying complex diseases.

## Supplementary Material

baae122_Supp

## Data Availability

All data described in this manuscript are available at https://disgenet.cn/.
