# Expression of Concern: DisGeNet: a disease-centric interaction database among diseases and various associated genes

**DOI:** 10.1093/database/baaf007

**Published:** 2025-01-31

This is an expression of concern regarding: Yaxuan Hu, Xingli Guo, Yao Yun, Liang Lu, Xiaotai Huang, Songwei Jia, DisGeNet: a disease-centric interaction database among diseases and various associated genes, *Database*, Volume 2025, 2025, baae122, https://doi.org/10.1093/database/baae122

Immediately following article publication, the journal was notified about significant similarities between the database described and an existing database, without attribution. While the editorial team investigates, please examine this study with care.

