# Supplementary material for: DisGeNet: a disease-centric interaction database among diseases and various associated genes
Source: Database (Oxford). 2025 Jan 11;2025:baae122. doi: 10.1093/database/baae122 (PMC11724190; doi:10.1093/database/baae122)
Supplement: baae122_Supp [file baae122_supp.zip › baae122_Supp/Supplementary File.doc]

# A detailed search example

1. Firstly, as shown in Figure 1, users can search disease/gene by disease name/gene ID, such as ‘AIDS’.

Figure 1 Keyword search in DisGeNet.

2. To accommodate potential user input errors, fuzzy search is supported in DisGeNet. As illustrated in Figure 2.1, up to the top 20 most similar results can be displayed in the search output. As shown in Figure 2.2, users can select the desired result to access more detailed information.

Figure 2 Partial search results. (1) Partial fuzzy search results. (2) Desires search result.

3. There are two forms, lists and graphs, to represent the results. Besides, as shown in Figure 3, users can click different buttons to perform various actions, such as linking to the view page, downloading relevant data and filtering node types.

Figure 3 The head of ‘List’ page. (1) Click to ‘View’ page. (2) Click to download link data about ‘AIDS’. (3) Click to filter node type.

3.1 In the list format, all interactions are presented with basic information. As illustrated in Figure 4, users can display all information, navigate to the result page of another node (e.g., hsa-miR-1-3p), and download subgraph data centered on another node (e.g., hsa-miR-1-3p) that is not the search node (i.e., not ‘AIDS’). Additionally, users can access relevant subgraph data and perform page redirection functionalities. By downloading subgraph data for ‘AIDS’ and its associated nodes, further analyses such as link prediction can be conducted.

Figure 4 List results of ‘AIDS’. (1) Click to display whole symbol of ‘AIDS’. (2) Click to turn the other node’s ‘List’ page. (3) Click to download subgraph data centered on another node. (4) Click to turn the page. (5) Input the target page number.

3.2 When users navigate to the view page via the 'View', as shown in Figure 5, detailed information about the node ('AIDS') is displayed. Users can further click the 'View' to display data graphs related to AIDS.

Figure 5 Head of ‘View’ page. (1) Click to detailed ‘View’ page.

3.3 As illustrated in Figure 6, DisGeNet provides statistical charts for node information, edge information, and the subnetwork graph. Users can move the cursor to specific positions to highlight and obtain detailed information. Additionally, users have the option to download the subnetwork graph.

Figure 6 Detailed ‘View’ page. (1) The statistic of node. (2) The statistic of link. (3) The subgraph about ‘AIDS’. (4) Click to restore or download the subgraph.
