# Supplementary material for: DisGeNet: a disease-centric interaction database among diseases and various associated genes
Source: Database (Oxford). 2025 Jan 11;2025:baae122. doi: 10.1093/database/baae122 (PMC11724190; doi:10.1093/database/baae122)
Supplement: baae122_Supp [file baae122_supp.zip › baae122_Supp/Supplementary Tables.doc]

**Supplementary Table 1.** A detailed investigation of the databases for various types of data within DisGeNet.

| Data type | Database | Description |
| --- | --- | --- |
| Disease | MONDO | MONDO is a comprehensive disease ontology database that integrates multiple disease data sources, offering detailed disease classification suitable for cross-database comparison and analysis. |
|  | DO | The Disease Ontology (DO) has been developed as a standardized ontology for human disease with the purpose of providing the biomedical community with consistent, reusable and sustainable descriptions of human disease terms, phenotype characteristics and related medical vocabulary disease concepts through collaborative efforts with biomedical researchers. |
|  | MeSH | Medical Subject Headings (MeSH) is the NLM controlled vocabulary thesaurus used for indexing articles for PubMed. |
|  | OMIM | Online Mendelian Inheritance in Man (OMIM) is a continuously updated catalog of human genes and genetic disorders and traits, with particular focus on the molecular relationship between genetic variation and phenotypic expression. |
|  | HPO | The Human Phenotype Ontology (HPO) provides a standardized vocabulary of phenotypic abnormalities encountered in human disease. |
|  | MedGen | MedGen organizes information related to human medical genetics, such as attributes of conditions with a genetic contribution. |
|  | GARD | GARD is a program of the National Institutes of Health that provides free access to reliable, easy-to-understand information about genetic and rare diseases. |
|  | MalaCards | MalaCards is a searchable, integrated database that provides comprehensive, user-friendly information on all annotated human maladies. |
| lncRNA | NCBI Gene | NCBI Gene integrates information from a wide range of species. A record may include nomenclature, Reference Sequences (RefSeqs), maps, pathways, variations, phenotypes, and links to genome-, phenotype-, and locus-specific resources worldwide. |
|  | GENCODE | GENCODE is a project that identifies and classifies all gene features in the human and mouse genomes based on biological evidence. |
|  | HGNC | The HGNC is responsible for approving unique symbols and names for human loci, including protein coding genes, ncRNA genes and pseudogenes, to allow unambiguous scientific communication. |
|  | NONCODE | NONCODE is an integrated knowledge database dedicated to non-coding RNAs (excluding tRNAs and rRNAs). |
|  | LNCipedia | LNCipedia is a public database designed to store long non-coding RNA (lncRNA) sequences and annotations. |
| protein-coding gene | NCBI Gene | NCBI Gene integrates information from a wide range of species. A record may include nomenclature, Reference Sequences (RefSeqs), maps, pathways, variations, phenotypes, and links to genome-, phenotype-, and locus-specific resources worldwide. |
|  | GENCODE | GENCODE is a project that identifies and classifies all gene features in the human and mouse genomes based on biological evidence. |
|  | HGNC | The HGNC is responsible for approving unique symbols and names for human loci, including protein coding genes, ncRNA genes and pseudogenes, to allow unambiguous scientific communication. |
| microRNA | miRBase | miRDB is an online database for miRNA target prediction and functional annotations. |
|  | TargetScan | TargetScanS predicts biological targets of miRNAs by searching for the presence of conserved 8mer and 7mer sites that match the seed region of each miRNA. |
| lncRNA-disease | Lnc2Cancer | Lnc2Cancer is a manually curated database that provides comprehensive experimentally supported associations between lncRNA or circRNA and human cancer. |
|  | RNADisease | RNADisease is an updated resource of RNA-associated diseases, providing RNA-disease analysis, enrichment and prediction . |
|  | lncRNADisease | LncRNADisease is a database that curates and predicts the roles of long noncoding RNAs (lncRNAs) in various diseases. |
|  | EVLncRNAs | EVLncRNAs is a comprehensive, manually curated, high-quality and freely accessible resource of integrated sequence, structure, functional and phenotypic information of exprimentally validated long noncoding RNAs from all species. |
|  | GeneCaRNA | GeneCaRNA is a comprehensive gene-centric database of human non-coding RNAs in the GeneCards suite. |
| microRNA-disease | HMDD | The Human microRNA Disease Database (HMDD) is a database that curated experiment-supported evidence for human microRNA and disease associations. |
|  | miR2Disease | miR2Disease , a manually curated database, aims at providing a comprehensive resource of miRNA deregulation in various human diseases. |
|  | miRCancer | miRCancer provides comprehensive collection of microRNA expression profiles in various human cancers which are automatically extracted from published literatures in PubMed. |
| protein-coding gene-disease | OMIM | OMIM is a comprehensive, authoritative compendium of human genes and genetic phenotypes that is freely available and updated daily. |
|  | HGMD | HGMD is a database of known gene lesions responsible for human inherited disease, maintained by the Institute of Medical Genetics in Cardiff. |
| microRNA-protein-coding gene | miRTarBase | miRTarBase has accumulated more than three hundred and sixty thousand miRNA-target interactions (MTIs), which are collected by manually surveying pertinent literature after NLP of the text systematically to filter research articles related to functional studies of miRNAs. |
|  | miRDB | miRDB is an online database for miRNA target prediction and functional annotations. |
|  | TarBase | TarBase constitutes the reference repository of experimentally supported miRNA targets on protein-coding transcripts. |
| protein-protein interaction | STRING | STRING is a database of known and predicted protein-protein interactions from various sources and organisms. |
|  | IntAct | IntAct is a database of molecular interactions curated from literature or direct data depositions. |
|  | BioGrid | BioGRID is a biomedical interaction repository with data compiled through comprehensive curation efforts. |
| microRNA-lncRNA | ENCORI | ENCORI is an openly licensed and state-of-the-art platform to facilitate the integrative, interactive and versatile display of, as well as the comprehensive annotation and discovery of, RNA-RNA and protein-RNA interactions by developing new software and pipelines to deeply mining thousands of high-throughput sequencing data of RNA-RNA interactome, CLIP-seq and degradome sequencing (degradome-seq). |
|  | MiRcode | MiRcode provides "whole transcriptome" human microRNA target predictions based on the comprehensive GENCODE gene annotation, including >10,000 long non-coding RNA genes. |
|  | LncACTdb | LncACTdb is a comprehensive database of experimentally supported ceRNA interactions and personalized networks contributing to precision medicine. |

**Supplementary Table 2. The information of diseases/genes collected in DisGeNet**

| Disease/Gene information | |
| --- | --- |
| pri_ID | The primary key of the data table |
| hgnc_ID | The HGNC ID of the gene |
| ensemble_ID | The Ensembl ID of the gene |
| entrez_ID | The NCBI GeneID of the gene |
| omim_ID | The OMIM ID of the gene and disease |
| miRbase | The accession of mature miRNA |
| mesh_ID | The Mesh ID of the disease |
| do_ID | The DO ID of the disease |
| name_for_spare1 | The NCBI gene name of the gene or the DO disease name of the disease |
| name_for_spare2 | The Ensembl name of the gene or the Mesh disease name of the disease |
| symbol | The symbol of the gene or the MONDO name of the disease |
| name | The gene name or disease OMIM name |
| alias | Other symbol of the gene or name of the disease |
| refseq_assession | The reference sequence |
| location | The sequence in genome |
| type | The type of the gene/disease |

**Supplementary Table 3. The information of associations/interactions collected in DisGeNet.**

| Association/Interaction information | |
| --- | --- |
| intera_ID | The ID of the link |
| interactor_A_ID | The ID of the first node |
| interactor_A_symbol | The symbol of the first node |
| interactor_A_alias | The alias of the first node |
| interactor_A_type | The type of the first node |
| interactor_B_ID | The ID of the second node |
| interactor_B_symbol | The symbol of the second node |
| interactor_B_alias | The alias of the second node |
| interactor_B_type | The type of the second node |
| method | The experimental method |
| PubMed_ID | The ID of the reference |
| pattern | The pattern of the link |
| source | The source of the database |
| intera_type | The type of the link |
| source_row_no | The number of rows in the database file |

**Supplementary Table 4. Results obtained through link prediction.**

| link | link type | method | literature |
| --- | --- | --- | --- |
| KRAS - non-small cell lung cancer | lncRNA-disease | Common Neighbors | Principe DR, Pasquinelli MM, Nguyen RH, et al. (2024) Loss of STK11 Suppresses Lipid Metabolism and Attenuates KRAS-Induced Immunogenicity in Patients with Non-Small Cell Lung Cancer. Cancer Res Commun., 4, 2282-2294. |
| LINC00963 - non-small cell lung cancer | lncRNA-disease | Common Neighbors | Xie Z, Zhong C, Shen J, et al. (2022) LINC00963: A potential cancer diagnostic and therapeutic target. Biomed Pharmacother, 150, 113019. |
| MIR17HG - non-small cell lung cancer | lncRNA-disease | Common Neighbors | Zheng G, Ye H, Bai J, et al. (2024) Downregulation of lncRNA MIR17HG reduced tumorigenicity and Treg-mediated immune escape of non-small-cell lung cancer cells through targeting the miR-17-5p/RUNX3 axis. J Biochem Mol Toxicol., 38, e23715. |
| NEAT1 - kidney cancer | lncRNA-disease | Common Neighbors | Liu R, Xu Z P, Li S Y, et al. (2022) BAP1-related ceRNA (NEAT1/miR-10a-5p/SERPINE1) promotes proliferation and migration of kidney cancer cells. Frontiers in Oncology, 12, 852515 |
